# Supplementary material for: Trends in Antidepressants Use in Spain between 2015 and 2018: Analyses from a Population-Based Registry Study with Reference to Driving
Source: Pharmaceuticals (Basel). 2020 Apr 3;13(4):61. doi: 10.3390/ph13040061 (PMC7243100; doi:10.3390/ph13040061)
Supplement: Supplementary file 1 [file pharmaceuticals-13-00061-s001.pdf]

Table S1: Antidepressants available in Castile and León (2015 – 2018).

Type of antidepressant	Code ATC	Name	DRUID categorization
Non-selective monoamine reuptake inhibitors	N06AA02	Imipramine	II
	N06AA04	Clomipramine	II
	N06AA06	Trimipramine	II
	N06AA09	Amitriptyline	III
	N06AA10	Nortiptyline	II
	N06AA12	Doxepine	III
	N06AA21	Maprotiline	II
Selective serotonin reuptake inhibitors	N06AB03	Fluoxetine	I
	N06AB04	Citalopram	I
	N06AB05	Paroxetine	I
	N06AB06	Sertraline	I
	N06AB08	Fluvoxamine	I
	N06AB10	Escitalopram	I
Monoamine oxidase A inhibitors	N06AG02	Moclobemide	I
Other antidepressants	N06AX03	Mianserin	III
	N06AX05	Trazodone	III
	N06AX11	Mirtazapine	III
	N06AX12	Bupropion	II
	N06AX14	Tianeptine	II
	N06AX16	Venlafaxine	II
	N06AX18	Reboxetine	I
	N06AX01	Duloxetine	II
	N06AX02	Agomelatine	II
	N06AX03	Desvenlafaxine	II
	N06AX06	Vortioxetine	II

Table S2: Evolution of the Castile and León population and drivers´s licences (2015 – 2018).

Population												
**Rank age**	**2015**			**2016**			**2017**			**2018**		
	**Male**	**Female**	**Total**	**Male**	**Female**	**Total**	**Male**	**Female**	**Total**	**Male**	**Female**	**Total**
0-4	45 405	42 504	87 909	44 382	41 386	85 768	42 905	40 144	83 049	41 604	39 121	80 725
5-9	50 925	48 078	99 003	50 665	47 821	98 486	50 035	47 344	97 379	48 594	45 657	94 251
10-14	49 439	47 220	96 659	49 847	47 730	97 577	50 316	48 259	98 575	51 124	48 407	99 531
15-19	48 620	46 904	95 524	48 862	46 935	95 797	48 939	46 706	95 645	49 610	47 885	97 495
20-24	54 724	53 382	108 106	53 230	52 333	105 563	52 182	51 246	103 428	51 428	50 777	102 205
25-29	62 787	61 247	124 034	61 109	59 382	120 491	59 522	57 531	117 053	58 298	56 506	114 804
30-34	75 089	71 664	146 753	71 742	68 841	140 583	68 575	66 051	134 626	65 942	63 241	129 183
35-39	90 372	87 031	177 403	87 267	83 676	170 943	83 600	80 400	164 000	79 663	76 944	156 607
40-44	92 686	89 879	182 565	92 967	90 094	183 061	92 799	89 681	182 480	92 434	89 499	181 933
45-49	93 082	91 643	184 725	93 035	91 392	184 427	92 076	90 588	182 664	91 744	89 952	181 696
50-54	93 252	90 618	183 870	93 251	91 395	184 646	93 500	91 893	185 393	92 913	92 426	185 339
55-59	87 280	84 212	171 492	88 988	85 894	174 882	89 831	86 956	176 787	90 852	87 988	178 840
60-64	72 448	69 337	141 785	75 073	72 029	147 102	77 520	74 875	152 395	79 640	77 583	157 223
65-69	65 430	66 777	132 207	66 403	67 268	133 671	67 615	68 053	135 668	67 660	67 825	135 485
70-74	56 526	61 968	118 494	58 076	63 396	121 472	58 913	64 067	122 980	60 320	64 908	125 228
75-79	45 154	56 939	102 093	43 540	53 807	97 347	43 510	52 990	96 500	46 205	55 251	101 456
80-84	44 543	62 354	106 897	44 319	62 772	107 091	42 312	60 175	102 487	39 465	56 065	95 530
85-89	27 547	46 335	73 882	28 618	47 555	76 173	29 407	48 337	77 744	29 731	48 690	78 421
≥ 90	13 282	30 034	43 316	14 119	31 809	45 928	14 662	32 970	47 632	15 333	34 407	49 740
**Total**	**1 168 591**	**1 208 126**	**2 376 717**	**1 165 493**	**1 205 515**	**2 371 008**	**1 158 219**	**1 198 266**	**2 356 485**	**1 152 560**	**1 193 132**	**2 345 692**

Driver´s licence census												
**Rank age**	**2015**			**2016**			**2017**			**2018**		
	**Male**	**Female**	**Total**	**Male**	**Female**	**Total**	**Male**	**Female**	**Total**	**Male**	**Female**	**Total**
15-19	9 282	5 586	14 868	9 238	5 634	14 872	8 357	4 689	13 046	8 702	5 102	13 804
20-24	43 294	35 387	78 681	42 165	34 280	76 445	40 859	33 207	74 066	39 837	32 570	72 407
25-29	55 831	50 618	106 449	53 617	48 755	102 372	51 913	46 861	98 774	50 269	45 329	95 598
30-34	69 810	61 387	131 197	66 192	58 489	124 681	62 677	56 134	118 811	59 765	53 560	113 325
35-39	86 841	75 838	162 679	83 112	72 880	155 992	79 204	70 129	149 333	74 693	66 771	141 464
40-44	89 294	76 277	165 571	88 673	76 856	165 529	88 025	76 717	164 742	87 163	76 513	163 676
45-49	90 151	74 310	164 461	89 641	74 389	164 030	88 611	74 242	162 853	87 961	74 534	162 495
50-54	90 450	67 282	157 732	90 290	69 036	159 326	90 158	70 695	160 853	89 420	72 062	161 482
55-59	85 820	56 346	142 166	87 607	59 781	147 388	88 543	62 443	150 986	89 826	64 796	154 622
60-64	71 450	36 255	107 705	74 219	40 173	114 392	76 748	44 338	121 086	79 194	48 281	127 475
65-69	62 572	23 964	86 536	63 824	26 068	89 892	65 577	28 466	94 043	65 978	30 748	96 726
70-74	51 161	12 390	63 551	52 595	13 755	66 350	53 663	15 096	68 759	55 094	16 442	71 536
75-79	35 993	5 000	40 993	34 852	5 277	40 129	35 960	6 008	41 968	38 829	7 105	45 934
80-84	28 304	1 941	30 245	28 194	2 055	30 249	27 809	2 319	30 128	26 500	2 491	28 991
85-89	14 160	429	14 589	14 133	484	14 617	14 548	588	15 136	14 814	661	15 475
≥ 90	2 944	22	2 966	5 669	50	5 719	6 176	77	6 253	7 350	112	7 462
**Total**	**887 357**	**583 032**	**1 470 389**	**884 021**	**587 962**	**1 471 983**	**878 828**	**592 009**	**1 470 837**	**875 395**	**597 077**	**1 472 472**

Table S3: List of the 40 DIMs more consumed in Castile and León into the study period (Packages/year).

Code ATC	Name	Packages	Code ATC	Name	Packages
N02BB02	Metamizole sodium	1 189 861	N05BA05	Potassium clorazeptae	160 627
N05BA06	Lorazepam	989 565	N05AH04	Quetiapine	158 143
N05BA12	Alprazolam	861 791	N06AA09	Amitryptiline	152 437
N02AJ13	Tramadol and paracetamol	683 935	N03AX14	Levetiracetam	130 749
N05CD06	Lormetazepam	582 253	N06AX05	Trazodone	130 569
N05BA08	Bromazepam	391 733	N02AX02	Tramadol	129 998
N03AX16	Pregabalin	277 073	N05AH03	Olanzapine	100 752
N06AB10	Escitalopam	274 420	N03AE01	Clonazepam	100 692
N02AJ06	Codeine and Paracetamol	273 087	N06AB04	Citalopram	96 353
N05BA01	DIAZEPAM	269 764	N06AX23	Desvenlafaxine	92 661
N05CF02	ZOLPIDEM	254 270	N06AB03	Fluoxetine	87 875
N06AB06	Setraline	244 838	N03AG01	Valproic acid	86 492
N06AX11	Mirtazapine	240 745	N04BA02	Levodopa and decarboxylase inhibitor	86 430
N06AX16	Venlafaxine	229 529	N05AL01	Sulpiride	84 765
A10BD07	Metformin and sitagliptin	212 812	A10BH01	Sitagliptine	83 896
N06AX21	Duloxetine	189 961	N03AX12	Gabapentine	83 492
N06AB05	Paroxetine	189 552	A10BB09	Gliclazide	82 833
A10AE04	Insuline glargin	185 578	A10BX02	Repaglinide	80 218
N02AB03	Fentanyl	182 729	N05AX08	Risperidone	79 294
A10BD08	Metformin and vildagliptin	179 957	A10BH05	Linagliptine	78 734

Table S4. Evolution of antidepressants use in Castile and León (2015 – 2018)

	Population % (95CI)				Drivers % (95CI)			
	**2015**	**2016**	**2017**	**2018**	**2015**	**2016**	**2017**	**2018**
**Antidepressants**								
Non-selective monoamine reuptake inhibitors								
Total	1.01 (1 - 1.02)	1.09 (1.08 - 1.1)	1.12 (1.11 - 1.13)	1.23 (1.22 - 1.24)	0.68 (0.67 - 0.69)	0.79 (0.78 - 0.8)	0.83 (0.82 - 0.84)	0.92 (0.9 - 0.94)
Male	0.51 (0.5 - 0.52)	0.57 (0.56 - 0.58)	0.58 (0.57 - 0.59)	0.65 (0.64 - 0.66)	0.53 (0.51 - 0.55)	0.59 (0.57 - 0.61)	0.61 (0.59 - 0.63)	0.69 (0.67 - 0.71)
Female	1.49 (1.47 - 1.51)	1.6 (1.58 - 1.62)	1.65 (1.63 - 1.67)	1.79 (1.77 - 1.81)	0.91 (0.89 - 0.93)	1.09 (1.06 - 1.12)	1.15 (1.12 - 1.18)	1.26 (1.23 - 1.29)
	X^2=50.326; p=0.001	X^2=48.125; p=0.001	X^2=39.478; p=0.001	X^2=52.147; p=0.001	X^2=1746.258; p=0.001	X^2=1896.324; p=0.001	X^2=1986.214; p=0.001	X^2=1896.247; p=0.001
Selective serotonin reuptake inhibitors								
Total	4.61 (4.58 - 4.64)	5.15 (5.12 - 5.18)	4.96 (4.93 - 4.99)	5.24 (5.21 - 5.27)	2.98 (2.95 - 3.01)	3.44 (3.41 - 3.47)	3.33 (3.3 - 3.36)	3.53 (3.5 - 3.56)
Male	2.43 (2.4 - 2.46)	2.79 (2.76 - 2.82)	2.66 (2.63 - 2.69)	2.86 (2.83 - 2.89)	2.45 (2.42 - 2.48)	2.77 (2.74 - 2.8)	2.67 (2.64 - 2.7)	2.86 (2.83 - 2.89)
Female	6.73 (6.69 - 6.77)	7.43 (7.38 - 7.48)	7.18 (7.13 - 7.23)	7.53 (7.48 - 7.58)	3.78 (3.73 - 3.83)	4.45 (4.4 - 4.5)	4.31 (4.26 - 4.36)	4.52 (4.47 - 4.57)
	X^2=985.254; p=0.001	X^2=1058.981; p=0.001	X^2=1125.698; p=0.001	X^2=974.251; p=0.001	X^2=6587.149; p=0.001	X^2=6418.187; p=0.001	X^2=6725.792; p=0.001	X^2=6658.217; p=0.001
Other antidepressants								
Total	3.14 (3.12 - 3.16)	3.81 (3.79 - 3.83)	3.73 (3.71 - 3.75)	4.17 (4.14 - 4.2)	1.96 (1.94 - 1.98)	2.43 (2.41 - 2.45)	2.4 (2.38 - 2.42)	2.66 (2.63 - 2.69)
Male	1.9 (1.88 - 1.92)	2.39 (2.36 - 2.42)	2.31 (2.28 - 2.34)	2.65 (2.62 - 2.68)	1.89 (1.86 - 1.92)	2.29 (2.26 - 2.32)	2.26 (2.23 - 2.29)	2.53 (2.5 - 2.56)
Female	4.34 (4.3 - 4.38)	5.18 (5.14 - 5.22)	5.11 (5.07 - 5.15)	5.65 (5.61 - 5.69)	2.08 (2.04 - 2.12)	2.63 (2.59 - 2.67)	2.61 (2.57 - 2.65)	2.83 (2.79 - 2.87)
	X^2=678.241; p=0.001	X^2=698.875; p=0.001	X^2=685.458; p=0.001	X^2=648.578; p=0.001	X^2=5420.369; p=0.001	X^2=5698.439; p=0.001	X^2=5324.946; p=0.001	X^2=5987.781; p=0.001

Abbreviations: 95CI. confidence interval

Table S5. Evolution of antidepressants use in Castile and León by DRUID classification (2015 – 2018)

	Population % (95CI)				Drivers % (95CI)			
	**2015**	**2016**	**2017**	**2018**	**2015**	**2016**	**2017**	**2018**
**DRUID clasiffication**								
**1**								
Total	4.63 (4.6 - 4.66)	5.17 (5.14 - 5.2)	4.98 (4.95 - 5.01)	5.26 (5.23 - 5.29)	2.99 (2.96 - 3.02)	3.46 (3.43 - 3.49)	3.34 (3.31 - 3.37)	3.54 (3.51 - 3.57)
Male	2.44 (2.41 - 2.47)	2.8 (2.77 - 2.83)	2.67 (2.64 - 2.7)	2.87 (2.84 - 2.9)	2.46 (2.43 - 2.49)	2.79 (2.76 - 2.82)	2.68 (2.65 - 2.71)	2.87 (2.84 - 2.9)
Female	6.76 (6.72 - 6.8)	7.46 (7.41 - 7.51)	7.21 (7.16 - 7.26)	7.56 (7.51 - 7.61)	3.79 (3.74 - 3.84)	4.47 (4.42 - 4.52)	4.32 (4.27 - 4.37)	4.53 (4.48 - 4.58)
	X^2=895.214; p=0.001	X^2=986.325; p=0.001	X^2=854.214; p=0.001	X^2=995.687; p=0.001	X^2=6358.214; p=0.001	X^2=5987.147; p=0.001	X^2=6398.257; p=0.001	X^2=5987.214; p=0.001
**2**								
Total	2.06 (2.04 - 2.08)	2.28 (2.26 - 2.3)	2.26 (2.24 - 2.28)	2.44 (2.42 - 2.46)	1.37 (1.35 - 1.39)	1.62 (1.6 - 1.64)	1.63 (1.61 - 1.65)	1.75 (1.73 - 1.77)
Male	1.15 (1.13 - 1.17)	1.3 (1.28 - 1.32)	1.3 (1.28 - 1.32)	1.41 (1.39 - 1.43)	1.23 (1.21 - 1.25)	1.38 (1.36 - 1.4)	1.4 (1.38 - 1.42)	1.5 (1.47 - 1.53)
Female	2.93 (2.9 - 2.96)	3.22 (3.19 - 3.25)	3.19 (3.16 - 3.22)	3.44 (3.41 - 3.47)	1.6 (1.57 - 1.63)	1.98 (1.94 - 2.02)	1.97 (1.93 - 2.01)	2.12 (2.08 - 2.16)
	X^2=70.289; p=0.001	X^2=65.365; p=0.001	X^2=69.587; p=0.001	X^2=67.354; p=0.001	X^2=1658.247; p=0.001	X^2=1587.965; p=0.001	X^2=1625.387; p=0.001	X^2=1710.256; p=0.001
**3**								
Total	2.42 (2.4 - 2.44)	3.03 (3.01 - 3.05)	3 (2.98 - 3.02)	3.41 (3.39 - 3.43)	1.48 (1.46 - 1.5)	1.87 (1.85 - 1.89)	1.87 (1.85 - 1.89)	2.11 (2.09 - 2.13)
Male	1.46 (1.44 - 1.48)	1.9 (1.88 - 1.92)	1.83 (1.81 - 1.85)	2.16 (2.13 - 2.19)	1.4 (1.38 - 1.42)	1.76 (1.73 - 1.79)	1.73 (1.7 - 1.76)	1.99 (1.96 - 2.02)
Female	3.36 (3.33 - 3.39)	4.11 (4.07 - 4.15)	4.13 (4.09 - 4.17)	4.62 (4.58 - 4.66)	1.59 (1.56 - 1.62)	2.04 (2 - 2.08)	2.08 (2.04 - 2.12)	2.29 (2.25 - 2.33)
	X^2=587.145; p=0.001	X^2=699.247; p=0.001	X^2=654.228; p=0.001	X^2=630.259; p=0.001	X^2=4458.698; p=0.001	X^2=4698.357; p=0.001	X^2=4968.587; p=0.001	X^2=4789.653; p=0.001

Abbreviations: 95CI. confidence interval
